# E1A oncogene induced sensitization to NK cell induced apoptosis requires PIDD and Caspase-2

**DOI:** 10.1038/s41420-019-0189-z

**Published:** 2019-07-01

**Authors:** Jay R. Radke, John M. Routes, James L. Cook

**Affiliations:** 1 Research Section, Boise VA Hospital and Idaho Veterans Research and Education Foundation, Boise, ID 83702 USA; 2 Section of Allergy and Clinical Immunology, Department of Pediatrics, Medical College of Wisconsin, Milwaukee, WI 53226 USA; 3 Research Section, Edward Hines, Jr. VA Hospital, Hines, Maywood, IL 60141 USA; 4 Division of Infectious Diseases, Department of Microbiology and Immunology, and the Infectious Diseases and Immunology Research Institute, Loyola University Chicago–Stritch School of Medicine, Maywood, IL 60153 USA

**Keywords:** Innate immunity, Apoptosis

## Abstract

Expression of the adenovirus E1A oncogene sensitizes tumor cells to innate immune rejection by NK cells. This increased NK sensitivity is only partly explained by an E1A-induced increase in target cell surface expression of NKG2D ligands. The post-recognition mechanisms by which E1A sensitizes cells to the apoptotic cell death response to NK injury remains to be defined. E1A sensitizes cells to apoptotic stimuli through two distinct mechanisms—repression of NF-κB-dependent antiapoptotic responses and enhancement of caspase-2 activation and related mitochondrial injury. The current studies examined the roles of each of these post-NKG2D-recognition pathways in the increased sensitivity of E1A-positive target cells to NK killing. Sensitization to NK-induced apoptosis was independent of E1A-mediated repression of cellular NF-κB responses but was dependent on the expression of both caspase-2 and the upstream, caspase-2 activating molecule, PIDD. Target cells lacking caspase-2 or PIDD expression retained E1A-induced increased expression of the NKG2D ligand, RAE-1. NK cell-induced mitochondrial injury of E1A-expressing cells did not require expression of the mitochondrial molecules, Bak or Bax. These results define a PIDD/caspase-2-dependent pathway, through which E1A sensitizes cells to NK-mediated cytolysis independently of and complementarily to E1A-enhanced NKG2D/RAE-1 ligand expression.

## Introduction

Through its ability to deregulate the cell cycle, E1A can immortalize mammalian cells when expressed after abortive infection or stable transfection, in a manner similar to other DNA tumor virus oncogenes and cellular oncogenes^1,2^. E1A immortalized cells are not tumorigenic in immunocompetent hosts, since E1A sensitizes cells to the apoptosis-inducing (and therefore tumor rejecting) activities of host immune effector cells, such as natural killer (NK) cells, cytotoxic T lymphocyte (CTL), and activated macrophages^3^. Data from several experimental animal models support the conclusion that such cellular immune defenses prevent tumor development by E1A-positive tumor cells. Newborn rats are more sensitive to tumor development by E1A-positive sarcoma cells than mature rats. The resistance of mature rats to tumor development by E1A-positive sarcoma cells is the result of increased NK activity in mature rats in comparison to newborn rats^4^. Studies comparing rejection of E1A-positive tumor cells by immunocompetent mice (T cell positive, NK cell positive), immunocompetent mice depleted of NK cells (T cell positive, NK cell negative), nude mice (T cell negative, NK cell positive), or CD3-ε transgenic mice (T cell negative, NK-negative) demonstrated the ability of the host NK cell response to reject E1A-expressing tumor cells in a T cell-independent manner^5^. Although the role of NK cells in rejection of E1A-positive tumor cells is well established, the cellular mechanisms through which E1A renders tumor cells sensitive to NK killing and tumor challenge rejection are incompletely defined.

NK killing of E1A-positive tumor target cells requires killer cell receptor interactions with NKG2D ligands in both human (MIC-A/MIC-B, ULBP) and murine tumor cell lines (RAE-1)^6^. The interactions of NKG2D and its ligands are required for efficient cytolysis of E1A-positive cells, as evidenced by blocking the interaction with blocking antibodies^6^. Furthermore, overexpression of RAE-1 in E1A-negative mouse tumor cells increases their susceptibility to NK cytolytic activity^6^. However, E1A-positive cells are still more susceptible to NK killing than E1A-negative/RAE-1-overexpressing cells, suggesting that enhanced RAE-1 expression is not the sole mechanism through which E1A renders cells sensitive to NK cytolytic activity^6^.

Cytolytic lymphocytes use two main mechanisms to kill target cells—perforin/granzyme and TNF/Fas mediated apoptosis—and E1A expression sensitizes cells to both of these mechanisms^7–10^. E1A modulates two cellular pathways to sensitize cells to apoptotic injury, depending on the nature of the injury^11,12^. E1A sensitizes cells to apoptosis induced through the extrinsic apoptosis pathway (triggered by TNF, Fas ligand, and TRAIL) by repressing transcription of NF-κB-dependent antiapoptotic gene expression^13–15^. We have also demonstrated that E1A sensitizes cells to apoptosis from intrinsic pathway injuries (chemotherapeutic agents and nitric oxide (NO)) through NF-κB-independent cellular mechanisms but is dependent on enhanced PIDD-mediated activation of caspase-2^11,12,16^.

We used rat NK cell killing of E1A-expressing target cells to determine the relative importance of NF-κB-dependent and NF-κB-independent cellular mechanisms of E1A-induced sensitivity to NK cytolytic activity. Rat NK cells are the most well characterized NK cells for systems testing the immunobiology and tumorigenicity of E1A-positive tumor cells. Rat NK can kill E1A-positive targets of mouse, rat, hamster, and human origin; the target cell killing is E1A expression level dependent, and there is a strong correlation between rat NK activity and ontogeny and in vivo rejection of E1A-positive tumor cells^4,17–20^. Furthermore, nude rat spleens can be used as a rich source of NK cells for assays such as those reported here, since the spleens contain approximately ten times the NK cytolytic activity of nude mouse spleens against cytolytic-susceptible target cells, such as mouse YAC-1 cells^4^. In situations where pure NK cell populations are required experimentally, the rat RNK-16 cell line can be used to reproduce nude rat spleen NK cell killing of E1A-positive target cells, and RNK-16 killing of mouse target cells is dependent on killer cell expression of NKG2D^19–21^.

Rat NK cell injury of E1A-expressing mouse cells was used in these studies to test the hypothesis that E1A-mediated sensitization to NK cytolysis is dependent on the PIDD–caspase-2 apoptotic pathway. Our results showed that NK cell-induced apoptosis of E1A-positive cells is independent of NF-κB cellular responses and that expression of caspase-2 and PIDD is required for NK-cell-induced apoptosis. The resistance of PIDD or caspase-2 knockdown cells to NK cell killing was independent of the level of target cell surface expression of RAE-1, the key NKG2D ligand in NK recognition of E1A-positive mouse cells, since cells with repressed PIDD and caspase-2 expressed a level of cell surface RAE-1 that was equivalent to that of parental E1A-positive cells that have normal levels of PIDD and caspase-2 expression. Collectively, these data indicate that E1A-induced sensitization to NK-mediated apoptosis occurs as the result of two independent but complementary E1A activities—increased recognition of E1A-positive cells by increased cell surface expression of NKG2D ligands, as reported previously, and enhanced activation of the PIDD–Caspase-2 apoptotic signaling pathway^6^, as defined in this report.

## Results

### NK cell mediated cytolysis of E1A-expressing cells is dependent on caspase activity

**Fig. 1:**
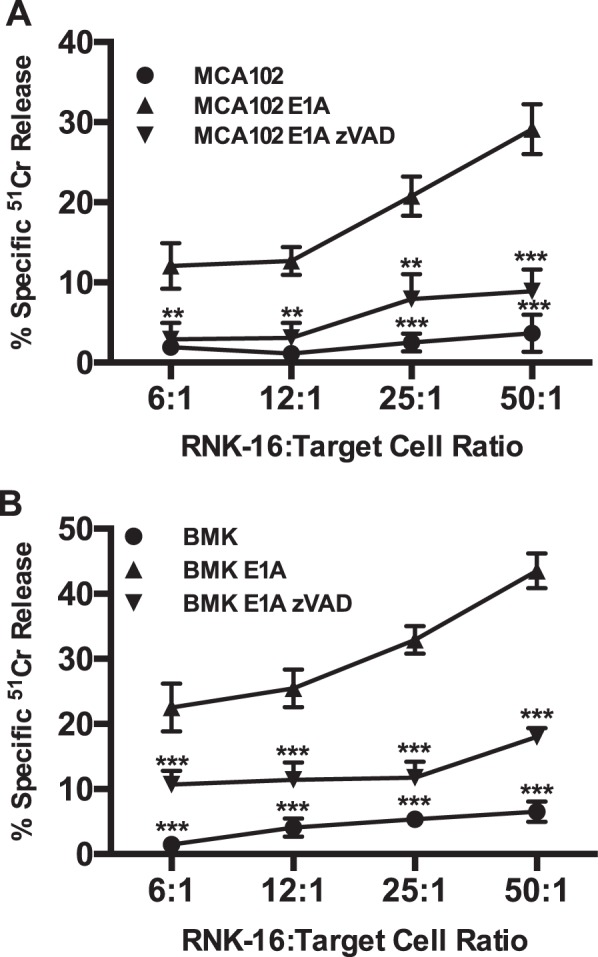
Role of caspases in NK-mediated cytolysis of E1A-expressing cells. **a** [Cr^51^]-labeled MCA102 (circle), MCA102 E1A (triangle) cells were incubated with RNK-16 cells at the indicated RNK:target ratios in the absence or presence (inverted triangle) of 100 μM zVAD-fmk. After 6 h, supernatants were collected and % specific Cr^51^ release was assessed (mean ± SEM; *n* = 4, ***P < 0.0001, **P ≤ 0.0084, one-way ANOVA). **b** [Cr^51^]-labeled BMK (circle) or BMK-E1A (triangle) cells were incubated with RNK-16 cells at the indicated RNK:target ratios in the absence or presence (inverted triangle) of 100 μM zVAD-fmk. After 6 h, supernatants were collected and % specific Cr^51^ release was assessed (mean ± SEM; *n* = 4, ***P ≤ 0.0001, one-way ANOVA)

Our laboratory has reported that E1A-expressing cells display both nuclear condensation and DNA fragmentation after incubation with cytolytic lymphocytes, indicating target cell death by apoptosis^22^. NK cells induce both caspase-dependent and -independent apoptotic pathways in target cells^23–25^. To determine if caspase activity is required for NK cell-induced apoptosis of E1A-positive target cells, we tested RNK-16 cell killing of E1A-positive MCA102 and BMK cells in the absence or presence of the pan-caspase inhibitor, zVAD (100 μM). Both E1A-positive cell types were highly susceptible to killing by RNK-16 cells, when compared with the relevant E1A-negative cells, and, in both cases, E1A-positive target cell killing was repressed in the presence of zVAD (Fig. 1a, b, respectively). Therefore, the cell death response to NK injury was caspase dependent.

### Target cell sensitivity to NK cell-induced apoptosis is not caused by E1A repression of NF-κB-dependent antiapoptotic defenses

**Fig. 2:**
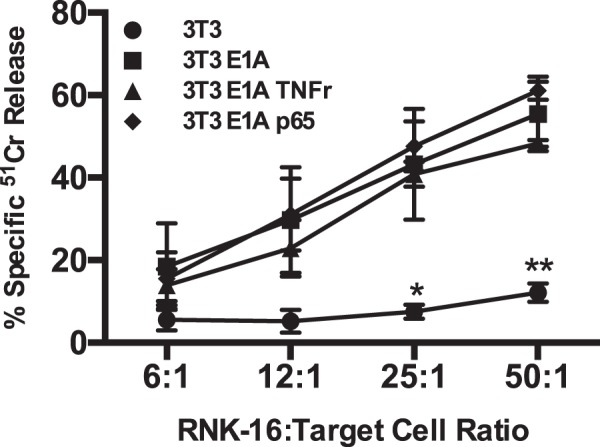
Role of NF-κB-dependent cellular responses in susceptibility of E1A-expressing cells to NK. [Cr^51^]-labeled NIH-3T3 (circle), 3T3-E1A (square), 3T3-E1A TNF resistant (TNFr) (triangle) or 3T3-E1A p65 RelA (p65) (diamond) cells were incubated with RNK-16 cells at the indicated RNK:target ratios. After 6 h, supernatants were collected and % specific Cr^51^ release was assessed (mean ± SEM; *n* = 3, **P = 0.0015, *P = 0.0126, one-way ANOVA)

To evaluate the role of E1A repression of NF-κB activation in sensitization to NK cell-induced apoptosis, we compared RNK-16 killing of NIH-3T3 cells stably transfected with E1A and known to be highly NK-sensitive (3T3 E1A) with two derivative 3T3 E1A cell lines created to be TNF resistant because of their NF-κB activation responses—3T3 E1ATNFr and 3T3 E1A p65^12^. Despite the high level, NF-κB-dependent resistance of these derivative cell lines to TNF, they were equally susceptible to killing by RNK-16 cells as parental, E1A-positive cells (3T3 E1A) (Fig. 2). The data in Fig. 2 indicate that the cellular mechanisms through which E1A sensitizes NIH-3T3 cells to NK killing are unrelated to E1A repression of NF-κB-dependent antiapoptotic effects.

### NK cells induce mitochondrial injury of E1A-expressing cells

**Fig. 3:**
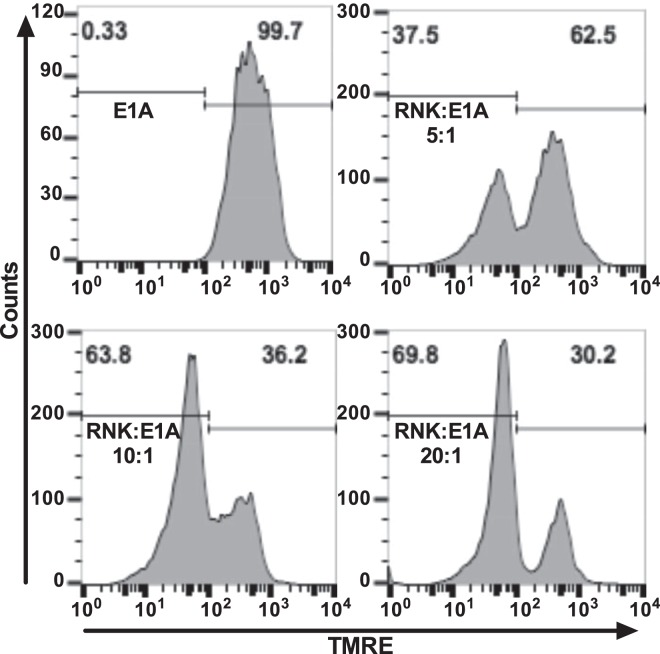
NK cell induced mitochondrial injury. 3T3-E1A cells were incubated with cell tracker blue-stained RNK-16 cells at the indicated ratios of RNK:E1A cells for 4 h and then stained with TMRE. Histograms show non-blue gated cells (3T3-E1A cells) and are representative of four individual experiments. Percentage of non-blue cells are shown above the markers

Our studies of other, NF-κB-independent apoptotic stimuli revealed that E1A enhances caspase-dependent, irreversible mitochondrial dysfunction after NO or chemotherapeutic drug injury. Therefore, we tested the NK cell cytotoxicity system, to determine whether E1A-positive cells exhibited a similar loss of mitochondrial function. RNK-16 cells were stained with cell tracker blue and then incubated in contact with 3T3 E1A cells for 4 h, after which mitochondrial membrane potential was assayed, using TMRE staining and flow cytometry (Fig. 3). A non-blue gate was used to assay TMRE staining of E1A-positive cells. The results showed that RNK-16 cells induced a loss of TMRE staining at NK cell:target cell ratios of 5, 10, and 20 to 1. The data presented to this point indicated that the mechanisms through which E1A sensitizes cells to NK killing involve both caspase activation (Fig. 1) and mitochondrial injury (Fig. 3).

### E1A-induced cellular sensitization to NK killing does not require Bak or Bax

The data in Fig. 3 indicated that NK killing of E1A-positive cells is associated with a marked loss of mitochondrial membrane potential. Such injury-induced mitochondrial dysfunction in cells destined for apoptotic death can be mediated by the mitochondrial effects of proapoptotic, Bcl-2 family members, including Bid, Bak, and Bax. Stimulus induced cleavage of Bid to its active form, tBid, triggers homodimerization of Bak and Bax, both of which alter mitochondrial membranes to cause loss of mitochondrial membrane potential and leakage of cytochrome *c* into the cytosol^26^. Granzyme B, produced by NK cells during cytolytic injury, can cause target cell mitochondrial depolarization and apoptosis both through this Bid/Bak/Bax pathway and through mechanisms that are independent of this pathway^27–29^.

**Fig. 4:**
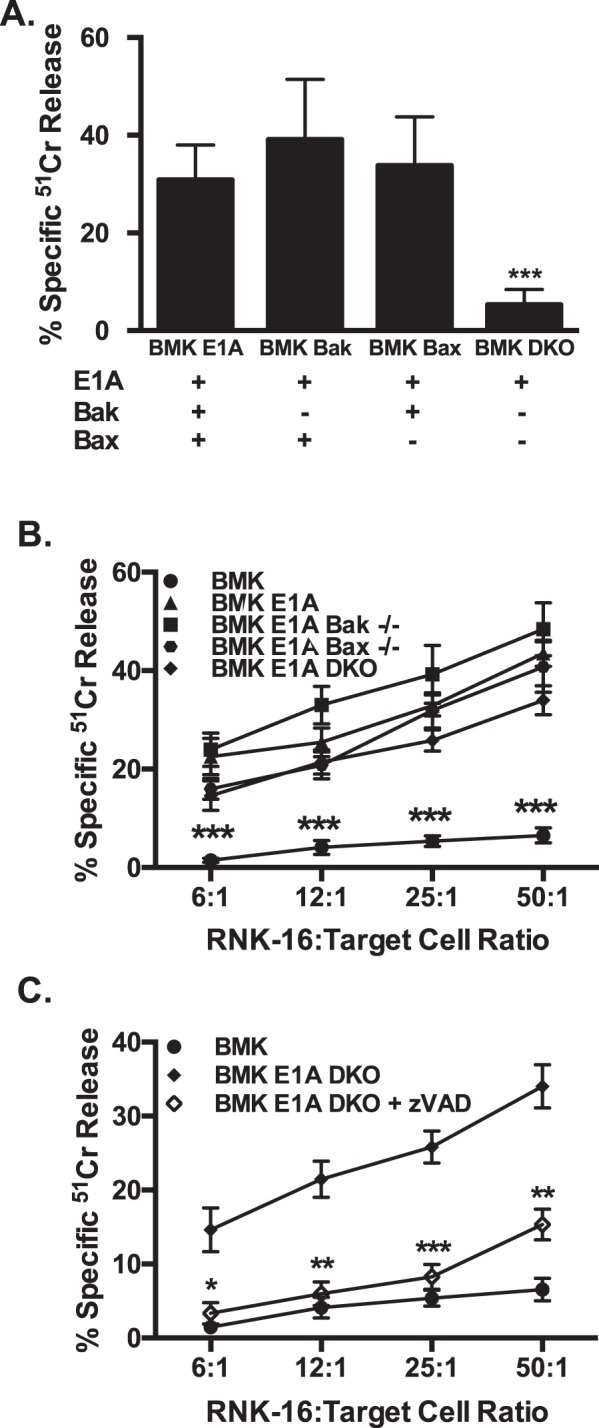
Role of Bak and Bax in RNK cytolysis of E1A-expressing cells. **a** [H^3^]-thymidine-labeled BMK-E1A, BMK-E1A-Bak^−/−^ (BMK Bak), BMK-E1A-Bax^−/−^ (BMK Bax), or BMK-E1A-Bak/Bax^−/−^/^−/−^ (BMK DKO) cells were incubated with ceramide (100 µM) overnight. Supernatants were collected and % specific thymidine release was assessed (mean ± SEM; *n* = 5, ***P = 0.001, one-way ANOVA). **b** [Cr^51^]-labeled BMK (circle), BMK-E1A (triangle), BMK-E1A-Bak^−/−^ (square), BMK-E1A-Bax^−/−^ (inverted triangle) or BMK-E1A-Bak/Bax^−/−^/^−/−^ (double knockout = DKO, diamond) cells were incubated with RNK-16 cells at the indicated RNK:target ratios. After 6 h, supernatants were collected and % specific ^51^Cr release was assessed (mean ± SEM; *n* = 4, ***P ≤ 0.0003, one-way ANOVA). **c** [Cr^51^]-labeled BMK (circle) or BMK-E1A-Bak/Bax^−/−^/^−/−^ (double knockout = DKO, diamonds) cells were incubated with RNK-16 cells at the indicated RNK:target ratios in the absence (filled diamond) or presence (open diamond) of 100 μM zVAD-fmk. After 6 h, supernatants were collected and % specific ^51^Cr release was assessed (mean ± SEM; *n* = 4, ***P ≤ 0.0001, **P = 0.0016, one-way ANOVA)

To determine whether E1A-induced target cell sensitization to NK cell-induced mitochondrial injury involves the Bid/Bak/Bax pathway, we obtained Bak, Bax, and Bak/Bax single and double knockout BMK cells that were transformed with E1A and dominant-negative mutant p53^30^. To confirm the role of Bak and Bax in the intrinsic apoptotic pathway in these cells, we treated wild type, Bak deficient (−/−), Bax deficient (−/−) or Bak/Bax double-deficient cells with ceramide. Ceramide triggers the intrinsic apoptotic pathway and results in mitochondrial injury that is mediated through Bak and Bax^31–34^. Wild type, Bak−/− and Bax−/− BMK cells were sensitive to ceramide-induced apoptosis, whereas Bak/Bax double-deficient cells were resistant (Fig. 4a). These results are similar to those reported with TNF-α and cycloheximide treatment^30^. As shown in Fig. 4b, cells expressing E1A and sufficient in Bak or Bax, deficient in either Bak or Bax or deficient in both Bak and Bax were equivalently sensitive to RNK-16 induced cytolysis. In the absence of both Bak and Bax, RNK-mediated apoptosis still required caspase activity (Fig. 4c). These data show that E1A enhancement of the intrinsic (mitochondrial injury) apoptosis pathway activated by NK cells is independent of Bid/Bak/Bax mechanisms.

### NK-mediated cytolysis of E1A-expressing cells requires Caspase-2 and PIDD expression

**Fig. 5:**
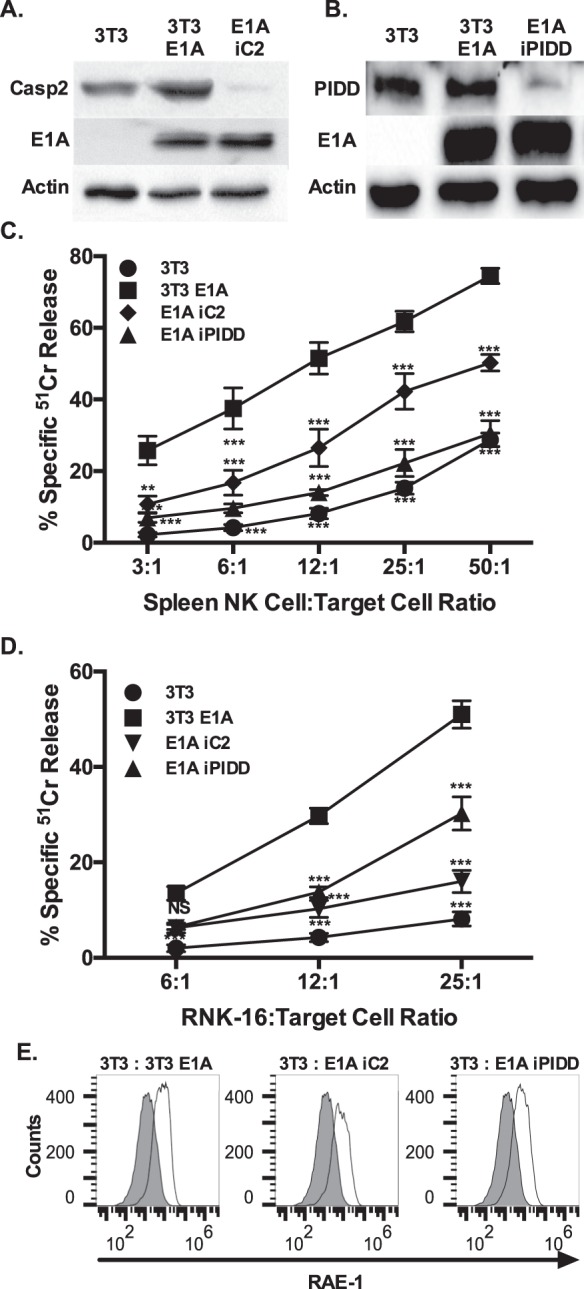
Role of caspase-2, PIDD and RAE-1 in the sensitivity of E1A-expressing cells to NK cell lysis. **a** Expression of Casp-2, E1A and actin in 3T3, 3T3-E1A and E1A-iC2 cell lines (originally published in^12^. **b** Expression of PIDD, E1A and actin in 3T3, 3T3-E1A and E1A-iPIDD cell lines^11^. **c** [Cr^51^]-labeled 3T3 (circle), 3T3-E1A (square), E1A-iC2 (inverted triangle), and E1A-iPIDD (triangle) cells were incubated with nude rat splenic NK cells at the indicated spleen cell:target ratios. % specific ^51^Cr release was assessed (mean ± SEM; *n* = 4, ***P ≤ 0.0002, **P = 0.0012, one-way ANOVA). **d** [Cr^51^]-labeled 3T3 (circle), 3T3-E1A (square), E1A-iC2 (inverted triangle), and E1A-iPIDD (triangle) cells were incubated with RNK-16 cells at the indicated RNK:target ratios. % specific ^51^Cr release was assessed (mean ± SEM; *n* = 4, ***P ≤ 0.0008, one-way ANOVA). **d** RAE-1 expression on 3T3 (filled histogram) compared to 3T3-E1A (open histogram), E1A-iC2 (open histogram), and E1A-iPIDD (open histogram) cells. Data shown are representative of four individual experiments

We have reported that E1A sensitization to intrinsic apoptosis, induced by both NO and etoposide, requires expression of both caspase-2 and its main activating platform member PIDD^11,12^. Both injuries induce caspase-dependent apoptosis and mitochondrial injury similar to what we observed with RNK-mediated injury of E1A-expressing cells in this study. We used E1A-positive, PIDD (E1A iPIDD), and caspase-2 (E1A iC2) shRNA knockdown cells to determine whether the PIDD–caspase-2 pathway is required for NK-mediated cytolysis of E1A-expressing target cells (Fig. 5a, b)^11,12^. Both types of knockdown cells were significantly less sensitive to lysis by RNK-16 NK cells (Fig. 5c) and nude rat splenic NK cells (Fig. 5d) than parental control 3T3 E1A cells, indicating that PIDD and caspase-2 are required for E1A sensitization to NK killing.

### Caspase-2 and PIDD-deficient cells retain increased RAE-1 expression

We have reported that E1A expression induces increased cell surface expression of the NKG2D ligands, RAE-1 (mouse cells) and MIC-A/B and ULPBP (human cells) and that this increased expression of NKG2D ligands on mouse or human cells results in an increased susceptibility to NK cell killing^6^. Rat NK cell killing of mouse tumor cells is dependent on the interaction of NKG2D and NKG2D ligands^21,35^. To exclude the possibility that selection of the clonally derived, E1A-positive caspase-2 (E1A iC2) and PIDD (E1A iPIDD) knockdown cells might have co-selected for cells with reduced NKG2D ligand expression, RAE-1 cell surface expression was examined by flow cytometry. Figure 5e shows that both E1A iC2 and E1A iPIDD cells retain the same level of cell surface expression of RAE-1 as the E1A-positive parental cells from which the clones were derived. E1A expression level in the E1A iC2 and E1A iPIDD cells was also comparable to that of the parental cells^11,12^. In addition to RAE-1, Mult-1 and H60 are other mouse NKG2D ligands. Expression of H60 was not enhanced in E1A-expressing cells (data not shown) and Mult-1 expression was also not altered by expression of E1A^6^, indicating that, in this model system, RAE-1 expression is the sole NKG2D ligand. In summary, these data indicated that E1A expression mediates increased susceptibility of target cells to NK killing through two independent and complementary pathways. The first is increased target cell recognition through increased cell surface NKG2D ligand (e.g., RAE-1) expression. The second is enhancement of the target cell proapoptotic response to NK cell injury, through the PIDD–caspase-2 pathway.

## Discussion

Expression of E1A sensitizes cells to the cytolytic machinery of NK cells. Previously, it has been shown that E1A expression results in increased expression of NKG2D ligands on the cell surface allowing for increased recognition by and increased sensitivity to lysis by NK cells^6^. However, increased expression of NKG2D ligand without E1A expression did not fully replicate the sensitizing effect of E1A^6^. The data presented here define PIDD–caspase-2 as the key, post-recognition cellular pathway through which E1A enhances target cell susceptibility to NK-cell-mediated cytolytic activity, as a complementary mechanism to E1A-induced increased expression of NKG2D ligands^6^. These results and our previously reported data also suggest that PIDD-dependent caspase-2 activation enhancement is a common cellular mechanism through which E1A sensitizes cells to a variety of stimuli of the intrinsic apoptosis pathway, including macrophage-NO and proapoptotic chemotherapeutic drugs^11,12,16^.

NK cells can kill target cells using multiple effector mechanisms, including production of TNF-superfamily members and transfer of cytolytic granules composed of perforin and granzyme at the NK cell/target cell synapse^36^. E1A utilizes two general mechanisms to sensitize cells to apoptotic cell death, depending on whether the proapoptotic stimulus triggers the extrinsic or the intrinsic apoptotic pathway. Sensitization to the extrinsic apoptotic pathway, triggered by members of the TNF superfamily, involves E1A repression of NF-κB-dependent transcription of antiapoptotic molecules^13,14,37,38^. The current data show that NK cells can also induce caspase dependent apoptotic cell death of E1A-expressing cells (Fig. 1) through mechanisms that are independent of E1A repression of NK-κB-dependent, antiapoptotic defenses (Fig. 2) but that mediate mitochondrial injury (Fig. 3). We have reported that induction of the intrinsic apoptotic pathway in E1A-expressing cells by NO and chemotherapeutic drugs requires PIDD-dependent activation of caspase-2 (refs. ^11,12,16^). The current studies extend that mechanistic pattern to NK cell killing of E1A-expressing target cells (Fig. 5c, d).

The multiple roles of caspase-2 in apoptosis remain to be completely defined. Caspase-2 can function as both an initiator and an effector caspase^39^. In its role as an initiator caspase, caspase-2 requires the formation of high molecular weight protein complexes, using the initiation platform called the PIDDosome^40^. The requirement, in our studies, for both PIDD and caspase-2 expression for efficient E1A sensitization of target cells to NK cell killing indicates that caspase-2 is functioning as an initiator caspase, during NK triggering of target cell apoptosis (Fig. 5c, d). These data and those published previously strongly suggest that E1A expression enhances caspase-2 activation through the PIDDosome in response to a variety of intrinsic apoptotic injuries^11,16^, including those mediated by NK cells.

Our observations provide insights into other possible cellular mechanisms through which E1A might enhance NK cell-induced, PIDD and caspase-2 dependent apoptosis of target cells. The short-term (6 h) NK assays used for these experiments reflect killing induced by cytolytic granules (composed of perforin and granzymes). Granzymes can cleave several cellular target molecules, including Bid, caspase-3, Hsp70, and Hsp90. Therefore, the question arises about whether E1A affects one or more of those granzyme-targeted cell molecule interactions in a way that enhances NK cell triggered PIDD–caspase-2 activation.

Previous studies have shown that granzymes can induce cleavage of Bid, which results in Bid mediated activation of Bax and Bak leading to mitochondrial injury^26,41–43^. Our data show that E1A-expressing cells remain sensitive to NK cell lysis in the absence of Bak and/or Bax (Fig. 4b), indicating that granzyme cleavage of Bid and subsequent Bak/Bax mediated mitochondrial injury are not required for E1A sensitization to NK cell-induced apoptosis. Our data on the lack of a role of Bcl-2 family members in modulating the susceptibility of E1A-expressing cells to NK killing are in agreement with those of others and ours that show that both NK and CTL can induce cell death of E1A-positive cells during expression of the adenoviral gene E1B 19K, which is a viral ortholog of Bcl-2 (refs. ^8,27,44,45^), which inhibits the proapoptotic effects of Bak and Bax^46^.

Cytolytic lymphocyte granzyme B can cleave capase-3, which can, in turn, cleave and activate caspase-2, thereby potentially bypassing the requirement for PIDD/PIDDosome activation of caspase-2 in the apoptotic response^47–49^. It is possible, therefore, that there might be both PIDD-dependent, initiator caspase-2 activation and PIDD-independent, caspase-3 enhanced downstream caspase-2 activation in E1A-positive target cells injured by NK cells. However, the marked reduction in NK susceptibility of E1A-positive, PIDD knockdown cells (E1A iPIDD; Fig. 5c, d)—almost to the level of cytolytic-resistant, E1A-negative cells—suggests that most of the E1A enhancing effect is PIDD-dependent and therefore at the level of initiator caspase-2 activity and not a downstream, post-caspase-3 effector caspase activity^50^.

Heat-shock proteins (Hsp70 and Hsp90) are PIDD chaperones that stabilize PIDD. Hsp90 binds full-length PIDD and is required for PIDD processing to both PIDD-C and PIDD-CC, the caspase activating form of PIDD^51^. Hsp70 and Hsp90 interact with PIDD, resulting in auto-proteolysis of PIDD from full-length to the active PIDD-CC form^52^. The presence of Hsp90 bound to PIDD-CC also acts as a negative regulator of caspase-2, by blocking the interaction of caspase-2 with PIDD-CC^53^. Therefore, the balance of Hsp interactions with PIDD and caspase-2 might determine the course of the cellular apoptotic response to a given stimulus. It is possible that NK cell granzyme activity and target cell E1A effects could affect this balance. Granzyme B can cleave Hsp70 and Hsp90 in target cells^54–56^. E1A induces increased expression of Hsp90 (ref. ^57^) and Hsp70 (ref. ^58^) and, therefore, might increase Hsp-mediated stabilization and processing of PIDD. Clarification of these intermolecular interactions among heat-shock proteins, PIDD, caspase-2, and E1A, in the context of NK cell injury of target cells, will require further study. Considering the variety of cellular molecular targets of E1A and granzymes that might impinge on the PIDD/caspase-2 activation process, it is not unlikely that there could be other mechanisms that might be involved in this E1A enhancing effect on the cellular apoptotic response.

E1A increases NKG2D ligand expression that causes increased target cell recognition by NK cells^6^. However, increased NKG2D ligand expression, in the absence of E1A, does not induce comparable target cell susceptibility to NK cell-induced killing and does not prevent tumor formation in mice^6^. These results suggest that there is a post-recognition mechanism through which E1A sensitizes cells to NK cell injury and that is independent of and complementary to effects on NKG2D ligand expression. In the current study, the two cell lines created to express reduced levels of either caspase-2 (E1A iC2) or PIDD (E1A iPIDD) retained the E1A-related increase in cell surface RAE-1 expression (Fig. 5e) but were significantly less susceptible to NK killing than the parental, E1A-positive cells from which they were derived (Fig. 5c, d). In summary, the data presented in this report support the conclusion that the E1A enhancing effect on the PIDD–caspase-2 activation response is the major post-recognition mechanism through which E1A enhances the target cell death response to NK cell injury. Considering the similar findings in our previous studies of NO and chemotherapy-induced apoptosis of E1A-expressing cells, we propose that E1A-mediated enhancement of PIDD-dependent activation of caspase-2 activity is the common pathway through which E1A sensitizes cells to stimuli that trigger the intrinsic apoptosis pathway.

## Methods and materials

### Cell lines and cell line characterization

The C57/B6-dervied, methylcholanthrene-induced sarcoma cell line, MCA102, was provided by Dr. N. Restifo (National Institutes of Health, Bethesda, MD) and maintained in Dulbecco's modified Eagle's medium (DMEM) with antibiotics and 5% fetal bovine serum (FBS)^59^. MCA102 E1A cells have been described^37^. E1A transformed baby mouse kidney cells (BMK) from wild type (wt; Bak+/+, Bax+/+), Bak−/−, Bax−/− and Bak−/− Bax−/− mice were obtained from Eileen White and maintained in DMEM with antibiotics and 10% FBS^30^. An NIH-3T3 cell line expressing Ad5 E1A 12S protein (MT12–1; herein referred to as 3T3 E1A) and its derivatives were maintained in DMEM supplemented with antibiotics and 5% calf serum^12,60^. The TNF-resistant, E1A-expressing NIH-3T3 cell lines, TR1 (herein referred to as 3T3 E1ATNFr) and A1 (herein referred to as 3T3 E1A p65), have been characterized^12^. These cell lines were derived from MT12–1/3T3 E1A cells, either through selection for survival in TNF (3T3 E1ATNFr) or through stable overexpression of the key NF-κB subunit, p65/RelA (3T3 E1A p65). 3T3-E1A+shRNA Caspase-2 cells (E1A-iC2) and 3T3-E1A+shRNA PIDD cells (E1A-iPIDD) have been characterized^11,12^. RNK-16 cells were provided by M. Nakamura (University of California at San Francisco) and were maintained in RPMI supplemented with 10% fetal bovine serum and β-mercaptoethanol^61^.

### Isolation of NK cells and cytotoxicity assays

Use of nude rats for isolation of spleen NK cells was approved by the Edward Hines, Jr VA Hospital IACUC committee. Rat splenic NK cells were isolated from 2-month-old nude rats as previously described with modifications^62^. Spleen cells were treated with ACK lysis buffer (Lonza) to lyse red blood cells. Resulting cells were resuspended in 2 ml RPMI+10% FBS and put on a nylon wool column for 1 h at 37 °C. Columns were washed with 10 ml of warm medium to elute the B-cell-depleted cell population. Aliquots of the resulting cell suspensions were stained with anti-CD161 (Clone 10/78 AbCam), >80% of the B-cell-depleted population was CD161+. ^51^Cr-release cytotoxicity assays were performed as described^63^.

### RNK-16 mediated effects on target cell mitochondrial membrane permeability

RNK-16 cells were stained with 80 μM Cell Tracker Blue (CTB) (Molecular Probes) and were incubated at the indicated ratios with 3T3-E1A cells in 24-well plates for 6 h at 37 °C. After 6 h, all cells were stained with 100 nM TMRE (Molecular Probes). All cells were collected with trypsin-EDTA and processed for flow cytometry on a BD LSR. A non-blue gate was used to separate 3T3-E1A cells from CTB-RNK-16 cells for analysis of mitochondrial membrane potential of the 3T3-E1A cells. CTB-stained RNK-16 cells retained equivalent cytolytic activity as non-stained RNK-16 cells.

### Extracellular RAE-1 staining

Cells were harvested in 2 mM EDTA-PBS and stained with either PE-conjugated monoclonal anti-mouse panspecific RAE-1 antibody or Rat IgG_2A_ isotype control (R&D Systems), and flow cytometry was performed on an Accuri C6.

### Statistical analysis

Graph Pad Prism 6 was used for statistical analysis. One-way ANOVA was first performed followed by post hoc analysis by the Holm–Sidak method to determine significance of differences between two specific data sets. For all analyses, *P* < 0.05 was considered a significant difference.
